# Decision-Level Fusion of Spatially Scattered Multi-Modal Data for Nondestructive Inspection of Surface Defects

**DOI:** 10.3390/s16010105

**Published:** 2016-01-15

**Authors:** René Heideklang, Parisa Shokouhi

**Affiliations:** 1 Division 8.5 Micro NDE, Federal Institute for Materials Research and Testing, Unter den Eichen 87, 12205 Berlin, Germany; 2 Department of Civil and Environmental Engineering, The Pennsylvania State University, 215 Sackett Bldg., University Park, PA 16802, USA

**Keywords:** multi-sensor data fusion, density estimation, scattered data, defect detection, nondestructive testing, registration errors

## Abstract

This article focuses on the fusion of flaw indications from multi-sensor nondestructive materials testing. Because each testing method makes use of a different physical principle, a multi-method approach has the potential of effectively differentiating actual defect indications from the many false alarms, thus enhancing detection reliability. In this study, we propose a new technique for aggregating scattered two- or three-dimensional sensory data. Using a density-based approach, the proposed method explicitly addresses localization uncertainties such as registration errors. This feature marks one of the major of advantages of this approach over pixel-based image fusion techniques. We provide guidelines on how to set all the key parameters and demonstrate the technique’s robustness. Finally, we apply our fusion approach to experimental data and demonstrate its capability to locate small defects by substantially reducing false alarms under conditions where no single-sensor method is adequate.

## 1. Introduction

Industrial nondestructive testing (NDT) refers to the inspection of materials, parts or structures concerning their condition without compromising their structural integrity. NDT experts employ different sensors depending on the material and the anticipated types of defects. Near-surface cracks represent one class of flaws that are commonly encountered in critical machine parts under dynamic loading such as turbine blades and bearings. Repeating cycles of dynamic stress promote the growth of invisible micro cracks into larger cracks that shorten the service life of the defective part. As such, early diagnosis of faults is critical to the operational safety and serviceability of a broad range of machinery. Among the NDT methods suitable for near-surface crack detection, special attention is paid to those that allow automatic data acquisition and provide accurate, quantitative and reproducible results in the form of digitized signals. Common candidate methods are ultrasonic testing (UT), eddy current testing (ET), magnetic flux leakage (MFL) testing, and thermal testing (TT). However, single-method inspection is often ambiguous. This is because each method reacts to changes in specific physical properties of the tested structure in the presence of a defect, but the intrinsic material and geometry properties of the structure may also produce similar and often indistinguishable changes in the recorded signals. For instance, MFL is affected by surface roughness and inhomogeneous magnetic properties, and TT’s high sensitivity to negligible manufacturing and handling marks and imperfections results in a multitude of false positive indications. These non-defect indications are referred to as structural noise, because unlike measurement noise, they are deterministic across repeated measurements. Yet, structural noise varies across different inspection techniques, whereas we hypothesize that actual flaws should be characterized by multi-sensor agreement. The underlying assumption is that all NDT sensors are in principle sensitive to the same class of defects, for example surface cracks in a certain parameter range with regards to their position, size and orientation. This concept lies at the heart of NDT data fusion. In other words, multi-sensor inspection differentiates flaw indications from structural noise, thus providing more reliable assessments.

Sensor fusion can be performed at various levels of signal abstraction [1], each with specific drawbacks and advantages. In particular, decision-level fusion deals with the higher level aggregation of data after individual detection. That is, each signal is first processed individually, and then fed into the fusion algorithm. We argue that decision-level fusion has several advantages over signal-level fusion for NDT inspection. First, unlike signal-level fusion, the data to be fused do not have to be interpolated at a common grid of positions. Although a spatial association step is still necessary to prepare individual detections for fusion, decision-level fusion can work on the un-interpolated signal intensities. As we will demonstrate, this approach allows for explicit handling of localization uncertainty, for instance due to registration errors. This is in contrast to signal-level fusion, where accurate registration is crucial [2,3] because errors can hardly be compensated. Strongly localized features, such as cracks, are particularly prone to misregistration and are therefore better handled at a higher level of signal abstraction. A further major benefit is the strategy’s modularity. The individual data collection and processing can be carried out independently by the respective experts to tailor the detection process specifically to each inspection method. One practical benefit of the modularity offered by decision-level fusion is that it allows combining individual results, even if fusion was not envisioned in the original testing plan. This also facilitates independence from the type of data source, making it possible to aggregate heterogeneous modalities ranging from manual inspection data to the output of fully automated scanning systems. Consequently, different sources of information can be effortlessly exchanged and the fusion strategy is readily adapted to unknown future changes of input sources.

Although there are recent studies in NDT proposing decision-level fusion, e.g., by weighted averaging [4], hypothesis testing [5], and Bayesian or Dempster-Shafer theory [6,7], all of these works still rely on image registration and interpolation to perform fusion at common grid points. In fact, we are not aware of any data fusion publication in the field of NDT dealing with scattered decision-level fusion, *i.e.*, using the original measurement locations, despite the aforementioned advantages.

We propose a new fusion strategy that combines spatially scattered detection locations to improve the detection performance compared to single-source methods. In this paper, we will address the problem of nondestructive surface inspection, although the methodology is quite generic and can be easily extended to the three-dimensional case of volume inspection. Our approach is detailed in Section 2 where the general idea is introduced using a simple example. Subsequently, we formalize the problem, describe our method and provide practical techniques for automatic parameter estimation. The developed approach is then applied to experimental multi-sensor NDT measurements by three different inspection methods. Finally, we quantitatively demonstrate the enhanced performance of multi-sensor crack detection using our technique over single-sensor inspection.

## 2. Methodology

**Figure 1:**
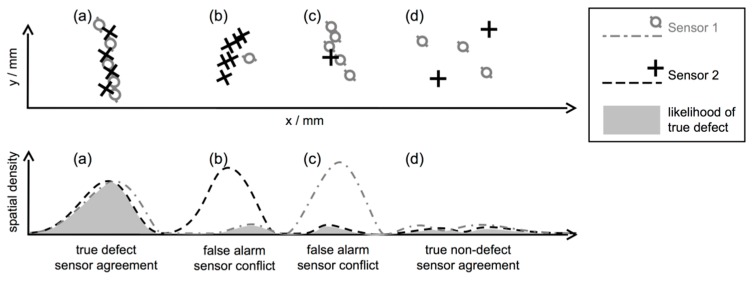
Schematic representation of the principle of the approach. The detection outcomes of two different NDT methods are represented by circles and crosses, for four cases (a)–(d). For each case, the corresponding spatial density per sensor along the *x*-axis is plotted below. The likelihood of observing a true defect (gray area) depends on both sensors yielding significant hit densities.

We will now schematically describe the core idea of our approach before giving a more formal definition. To this end, assume that information about potential defect locations $d=\left(d_{x},d_{y}\right)$, called *hits* in the following, was obtained from different NDT data sets. For example, consider Figure 1 for an outcome of individual surface inspection using two NDT methods. In cases (a)–(d), each dot marks a hit generated by some detection rule per sensor. Among the hits, there are also false alarms, for instance indicating changes in material properties unrelated to a defect (structural noise). Such false alarms are illustrated by cases (b) and (c) in Figure 1. Using single-sensor inspection, these false alarms cannot be distinguished from indications produced by actual defects such as those shown in case (a). A multi-sensor data set, on the other hand, is able to reveal case (a) as a real defect by assessing the agreement among different detection methods. Here, agreement is expressed in terms of joint spatial density of hits, taking into account all sensors. Our rationale is that the joint hit density is higher over real defects than in other areas, provided sufficient signal to noise ratio (SNR) for at least two sensors. On the contrary, a clear conflict occurs where only one sensor generates hits, and thus the joint density is not significantly increased relative to the individual sensor density. This concept is depicted at the bottom of Figure 1, where for each sensor the spatial density of hits across the specimen surface is symbolized. Only in case (a) both sensors agree in increased spatial density, whereas in cases (b) and (c) the sensors do not agree. Although there is also agreement in case (d) as well, the joint density is not significant enough, indicating the low likelihood of defect presence. This example demonstrates the potential of the joint spatial density as the basic mechanism for multi-sensor detection.

**Figure 2:**
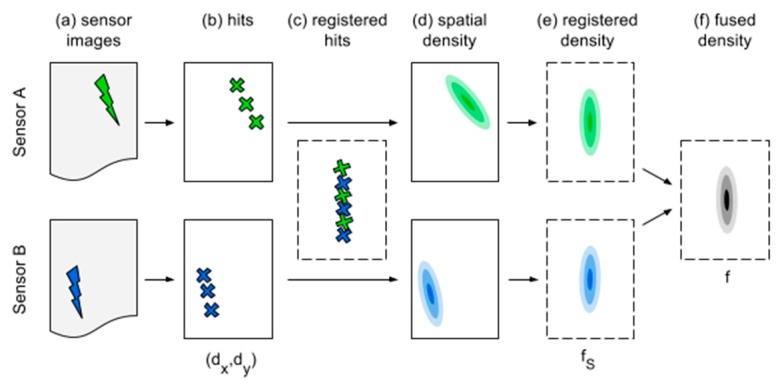
Flow chart of the fusion process. Gray boxes denote original sensor images, each containing an indication (crack symbol). Solid-edge boxes denote local (per sensor) coordinate systems; dashed boxes denote the global (registered) coordinate system. Cross markers denote hits. Two-dimensional spatial densities are indicated by contour plot symbols. Mathematical symbols below the boxes correspond to the notation used throughout this work. (**a**) Sensor images; (**b**) Hits; (**c**) Registered hits; (**d**) Spatial density; (**e**) Registered density; (**f**) Fused density.

We propose evaluating the local hit density as a measure for multi-sensor data fusion at decision level. Figure 2 provides a flow chart of the individual steps. The first step consists of generating hypothesized defect positions from individual NDT images. Additionally, each hit is associated with its local signal to noise ratio, which will be used later as a weighting factor (not shown in the flow chart). Suitable detection techniques depend on the respective NDT modality, but often a simple threshold operation on the sensor intensities is appropriate. It is important to make the detection strategies focus on sensitivity rather than specificity, thus ensuring that all (unknown) real defect indications are retained for the final detection by fusion. At the same time, we would still want to discard as many false alarms as possible. The difficulty of facing this trade-off, which is typical for single-sensor detection, is however less critical for multi-sensor detection because the main work is done by the subsequent fusion procedure. Practical examples of single-sensor detection will be given in Section 3.2.

The extracted hit locations from different NDT techniques are usually not expressed with respect to the same coordinate system. This is because rather than measuring at the very same locations on the specimen, it is often more practical to mathematically register the individual local coordinate systems after the measurements were carried out. Therefore, for each sensor pair, we find corresponding locations in the sensor data and fit a coordinate transformation model that minimizes the distance between each pair of corresponding locations. Note that, in contrast to fusion at the signal level, this transformation is not used here to interpolate the sensor data values. Rather, for decision-level fusion, the transformations allow to express the locations of the individual hits in a common coordinate system. We note that although each NDT method usually uses gridded measurement positions, the mapped hit locations after registration are not jointly gridded in the common coordinate system, but appear scattered instead. This is visualized in Figure 2c.

After establishing relationships between the individual coordinate systems, the next question is how to implement the density-based fusion concept already introduced before. One central challenge in the decision-level fusion of non-gridded locations is the uncertainty in localization. Two main factors contribute to this uncertainty. First, each sensor’s localization ability is limited by the physical resolution as well as the spatial sampling rate. Second, the coordinate transformations computed during spatial registration might be inaccurate to some degree. To be robust, a fusion approach must adequately cope with the inherent uncertainties about hit positions and must associate nearby hits for the purpose of density quantification. This loose concept of “proximity” should therefore be mathematically formalized.

To that end, various non-parametric techniques have been developed such as Mean shift [8], DBSCAN [9], OPTICS [10], Spectral clustering [11], and Kernel density estimation (KDE) [12,13]. We select the framework of kernel density estimation. This choice was motivated by considering that our data space typically has only two or three spatial dimensions, independent from the number of sensors. Therefore, the density can be directly modeled without being affected by the curse of dimensionality. Furthermore, it allows us to evaluate the density at arbitrary positions, not only at the hits.

Returning to the flow chart, KDE is applied to estimate the spatial hit densities from each individual sensor in Figure 2d,e to associate nearby hits. Finally, Figure 2f consists of a fusion rule that combines the individual densities and produces a fused image in which higher intensity corresponds to increased estimated likelihood of defect presence.

Next, we will formally introduce our method using ideas from KDE, and propose different fusion rules that implement the behavior of the gray shaded area in Figure 1 to recognize conflicts and agreement among the densities of different sensors.

### 2.1. Kernel Density Estimation (KDE)

Before proposing our technique, we repeat fundamental concepts of KDE in this section. These concepts and the associated notation are adopted in the rest of the paper.

KDE is a nonparametric statistical method to estimate a probability density function $\hat{f}$ from a set of samples $x_{i}$. The result is a continuous function, computed from a weighted sum of kernel functions $K_{h}$ with an associated bandwidth h, each centered over one of the samples: $\hat{f}\left(y\right)={\left(\sum_{i}w_{i}\right)}^{-1}\sum_{i}w_{i}K_{h}\left(y-x_{i}\right)$, with $K_{h}\left(x\right)=\frac{1}{h}K\left(\frac{x}{h}\right)$. Some functions qualifying as a kernel K are the uniform, triangle, Gaussian or Epanechnikov kernel functions. The bandwidth h controls the size of the neighborhood in which samples influence the density at a specific location. The choice of the bandwidth is critical for the overall performance of the algorithm. If the bandwidth is chosen too wide, KDE results in an overly smoothed density, thus losing important details of the distribution. On the other hand, if it is chosen too narrow, the estimate adapts too much to the specific realization of the sample set, thus missing the global features of the density. This problem has been well-studied, and several solutions have been proposed [14]. We will describe how to automatically compute a suitable bandwidth for our problem in Section 2.2.2.

The general formulation given above for KDE includes the normalization constant ${\left(\sum_{i}w_{i}\right)}^{-1}$, ensuring that the density integrates to one. Since we do not require a probabilistic interpretation, we proceed with a simpler unnormalized version of KDE by dropping the normalization constant. This also simplifies the notation. Furthermore, since the density estimate is a weighted sum with one term per data point, the data set can be partitioned to aggregate the total density function $\hat{f}$ from the sub-densities ${\hat{f}}_{J}$, each including only the samples $x_{i}$ from partition j:

(1) $$\hat{f}\left(y\right)=\underset{j}{\sum }{\hat{f}}_{J}\left(y\right)=\underset{j}{\sum }\underset{i\in P_{j}}{\sum }w_{i}K_{h}\left(y-x_{i}\right)$$

$P_{j}$ denotes a subset of all points such that partitions do not overlap and the union of all partitions covers the complete data set. This re-arrangement is taken up in the following section to group hits by each sensor, as is illustrated in Figure 2d.

KDE can be extended to vector-valued samples. To that end, let $x_{i}$ denote the ith vector-valued (bold face) sample, and let y denote the vector-valued location where to evaluate the density. Multivariate KDE is computed from multivariate kernel functions and an associated bandwidth matrix H, which describes the scale and the orientation of the kernels. A special kind of multivariate kernel is the product kernel, defined by $K_{H}\left(x\right)=\prod_{j}\frac{1}{h_{j}}K\left(\frac{x_{j}}{h_{j}}\right)$, where one univariate kernel for each dimension j is evaluated. The $h_{j}$ are the entries of the diagonal bandwidth matrix, *i.e.*, product kernels are not arbitrarily oriented in the data space. This property reduces the computational demand, because the data dimensions are considered independently. We use product kernels in our approach, which is described next.

### 2.2. Scattered Decision-Level Fusion

#### 2.2.1. Overview

**Figure 3:**
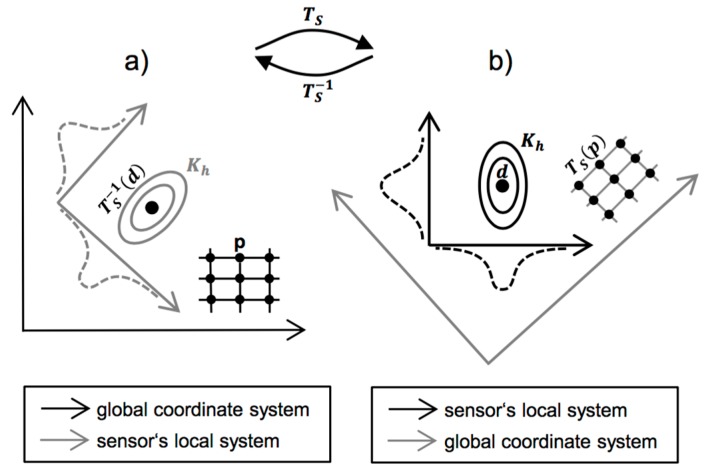
Coordinate transformation during the computation of the fused density. The two black coordinate systems in (**a**,**b**) are related through the coordinate transform $T_{s}$. (**a**) Coordinate system in which the fused density will be evaluated at gridded points p . The coordinate systems of the individual sensors, where the hits d are defined, are not axis-aligned with the global system. In particular, the kernels $K_{h}$ would require non-diagonal bandwidth matrices; (**b**) Coordinate system of one of the sensors, given by its measurement grid. For single-sensor density estimation, kernels $K_{h}$ are axis-aligned to the sensor’s system, thus facilitating product kernels. The single-sensor density is then evaluated at the transformed points $T_{S}\left(p\right)$.

In this section, we develop a new fusion method for our NDT problem based on concepts from KDE. Here, the role of the vector-valued data sample $x_{i}$ is taken by the two-dimensional hit location d as detected by a single sensor $S_{i}$ during surface inspection. As defined in Equation (1), the *joint density* that includes the hits from all sensors can be computed from *partial densities* that include only the hits from a single sensor. We extend this property to a more general framework of density-based fusion. Specifically, we introduce two modifications: (1) Each partial density is computed in the respective local coordinate system, using sensor-specific KDE parameters; and (2) To merge the partial densities into the joint density, the outer sum from Equation (1) is generalized to an arbitrary fusion rule F. Our approach is outlined by the following steps:

1. Define a grid of discrete locations ***p*** where the fused density should be evaluated. These locations are defined in the common coordinate system after registration, so that they refer to the same location on the specimen for all individual inspection methods. Map these locations to each local coordinate system using the coordinate transforms *T_S_* obtained during spatial registration. See Figure 3 for an illustration of this step.
2. For each individual sensor, compute the spatial density $\hat{f_{s}}(p)$ of single-sensor hits d and evaluate the density at the mapped locations $T_{S}\left(p\right)$:
  (2) $${\hat{f}}_{S}\left(p\right)=N\left(h_{s},\Delta^{S}\right)\underset{d\in D\left(S\right)}{\sum }w_{d}K_{h_{S}}\left(T_{S}\left(p\right)-d\right)$$
  The normalization constant $N\left(h_{S},\Delta^{S}\right)$ and the bandwidth $h_{S}$ are sensor-specific parameters. N depends on the kernel bandwidth and on the sampling distances $\Delta^{S}=\left(\Delta_{x}^{S},\Delta_{y}^{S}\right)$ (pixel dimensions). $w_{d}$ is a per-hit weighting factor.
3. For each evaluation point p, fuse the partial density values ${\hat{f}}_{S}\left(p\right)$ from the different sensors using a fusion rule *F*:
  (3) $$\hat{f}\left(p\right)=F\left(\left\{{\hat{f}}_{S_{1}}\left(p\right),\ldots ,{\hat{f}}_{S_{N}}\left(p\right)\right\}\right)$$

We will now provide a detailed explanation of these steps. The grid defined in Step 1 determines the resolution at which the fused density will be sampled. The grid size depends on the kernel bandwidths, because smoother densities computed from larger kernels facilitate coarser sampling grids to reduce the computational complexity. For optimal resolution however, we propose setting the grid size according to the sampling distance of the finest-sampled individual sensor. In the second step, the partial densities are computed as explained next.

.

#### 2.2.2. Estimation of Partial Densities

As noted, our first modification to standard KDE is to carry out density estimation in a per-sensor manner. To this end, the computations in Step 2 are defined in the respective local system for each sensor. Consequently, the kernel function $K_{S}$ can be defined as an axis-aligned product kernel to reduce the computational cost. The corresponding bandwidth parameters $h_{S}$ are chosen based on background knowledge about the nature of our data. Intuitively, the density estimator should always be able to smoothly interpolate between neighboring hit locations. For NDT data, the smallest possible distance between any two hits of the same sensor is given by the known spatial sampling intervals. For a measurement grid per sensor S, the two spatial sampling distances are denoted by $\Delta_{x}^{S},\;\Delta_{y}^{S}$ in the sensor’s coordinate system. To ensure that neighboring line scans crossing the same defect do not form disconnected density peaks, the kernel functions should at least stretch across one pixel in the sensor image. However, to avoid merging two unrelated indications, the kernels should not be made much larger. Therefore, we propose to use product kernels with minimum bandwidth parameters of $h_{s}=\left(h_{x},h_{y}\right)=\left(\Delta_{x}^{S},\Delta_{y}^{S}\right)$ for each sensor. It is natural to use product kernels for gridded individual measurements, because the bandwidths directly correspond to the physical pixel dimensions.

The aforementioned kernel size is a minimal setting. In practice, the most significant factor contributing to the localization uncertainty may not be the spatial sampling, but inevitable registration errors. The kernel sizes should be set large enough to smooth out these unwanted variations and to associate poorly registered indications. As a general approach, we propose the following kernel size:

(4) $$h_{s}=\left(h_{x},h_{y}\right)=\frac{\hat{u}}{min\left\{\Delta_{x}^{S},\Delta_{y}^{S}\right\}}\left(\Delta_{x}^{S},\Delta_{y}^{S}\right)$$

where $\hat{u}$ denotes an estimate of the localization uncertainty, for instance the mean registration error. This formulation keeps the kernel size ratio $h_{x}/h_{y}=\Delta_{x}^{S}/\Delta_{y}^{S}$ fixed, and scales the two-dimensional kernel size proportionally to $\hat{u}$. Consequently, in the fine-sampled direction corresponding to $min\left\{\Delta_{x}^{S},\Delta_{y}^{S}\right\}$, the kernel will be exactly $\hat{u}$ wide. In the other direction, the kernel is larger to maintain the ratio. Note that with increasing kernel size, the advantage of having spatially accurate sensors may be lost. Also, closely situated defects become harder to separate. Therefore, fusion performance benefits from high-quality registration by facilitating narrow kernels.

Setting the kernel size according to the localization uncertainty implies that sensors with fine spatial sampling produce more hits in the area of influence of a kernel than sensors with coarse sampling. To prevent finely-sampled sensors from having more influence on the fusion process by contributing larger densities, normalization is required. To this end, we define the normalization factor from Equation (2):

(5) $$N\left(h_{S},\Delta^{S}\right)=1/max\left\{\frac{h_{x}}{\Delta_{x}^{S}},\frac{h_{y}}{\Delta_{y}^{S}}\right\}$$

This essentially normalizes with regard to the number of pixels per kernel size, which implicitly relates to the potential number of hits in each dimension. Note that if the kernel size $h_{S}$ is defined according to Equation (4), then we have $N\left(h_{S},\Delta^{S}\right)=1/max\left\{\frac{h_{x}}{\Delta_{x}^{S}},\frac{h_{y}}{\Delta_{y}^{S}}\right\}=\Delta_{x}^{S}/h_{x}=\Delta_{y}^{S}/h_{y}=min\left\{\Delta_{x}^{S},\Delta_{y}^{S}\right\}/\hat{u}$.

In our method, the per-sensor normalization factor N replaces the conventional kernel normalization factor for product kernels, $N\left(h_{S}\right)=1/\left(h_{x}\cdot h_{y}\right)$.

Although the previous considerations are valid for all types of kernel functions, we suggest using a compactly supported kernel function like the *Epanechnikov product kernel* as defined in Equation (6). The compact support has the advantage of limiting the spatial influence area of each hit, which is expressed by the kernel bandwidth parameters $h=\left(h_{x},h_{y}\right)$, and thus facilitates faster computation than e.g., the non-vanishing Gaussian kernel:

(6) $$K_{h}(v)=\{\begin{array}{c} (1-{\left(\frac{v_{x}}{h_{x}}\right)}^{2})(1-{\left(\frac{v_{y}}{h_{y}}\right)}^{2}),|v_{x}|\leq h_{x}\;\text{and}\;|v_{y}|\leq h_{y}\\ 0,else \end{array}$$

To further adjust the quantification of density, we scale the kernel functions according to the weight $w_{d}$ per hit; see Equation (2). These weights control the influence of each hit on the final KDE. We set each weight proportional to the hit’s signal to noise ratio, so that clear indications have more impact on the final density than insignificant ones. Also, the weights offer additional flexibility to regulate the fusion result with regards to specific sensors or different inspection areas.

#### 2.2.3. Fusion of Partial Densities

In Equation (3) a fusion rule F is introduced that combines the partial densities. The most basic fusion rule is to sum up the individual densities, which in effect computes the total kernel density according to Equation (1). However, this approach has a major drawback concerning false alarms. High-intensity single sensor contributions have a large effect on sums, even when such indications are not backed up by other sensors. As an extreme example, the *maximum* function is most prone to false alarms. Therefore, more conservative rules are required to capture the agreement among sensors for effective reduction of false alarms. The following fusion rule is conceptually similar to the *sum* rule as used in conventional density estimation, but unlike the *sum* it guards against single-sensor false alarms:

(7) $$F_{sumIgnoreMax}\left(\left\{{\hat{f}}_{S_{1}}\left(p\right),\ldots ,{\hat{f}}_{S_{N}}\left(p\right)\right\}\right)=\left(\underset{S\in \left\{S_{1},\ldots S_{N}\right\}}{\sum }{\hat{f}}_{S}\left(p\right)\right)-\underset{S\in \left\{S_{1},\ldots S_{N}\right\}}{max}{\hat{f}}_{S}\left(p\right)$$

Note that the maximum is evaluated separately for each evaluation point p. Equation (7) realizes the quantification of agreement among sensors, because a large fused score now requires at least two sensors to produce high individual densities. Thus, $\hat{f}$ is expected to behave similarly to the function indicated by the shaded area in Figure 1. Note that in the case of only two available sensors for fusion, subtracting the maximum is equal to the *minimum* fusion rule, which is a fuzzy *AND*-operator, and is in fact the operation used to generate the shaded area in the figure. However, as more than two sensors are involved, requiring that all sensors indicate a defect might be too strict for some applications. Therefore, ignoring the maximum contribution can be viewed as a much milder version of the *AND* fusion rule. Other fusion rules that conceptually differ more from conventional density estimation, but also suit the quantification of agreement, are the *median*, *harmonic mean*, *geometric mean* and the *product.* An example of a more sophisticated rule is presented in [15], where the authors developed a Bayesian fusion approach that explicitly models inconsistencies among sensors, such as false alarms. However, we focus on simple algebraic fusion rules in this study to avoid introducing additional parameters by more complex methods.

In total, our fusion approach includes three mechanisms to ensure robustness against false alarms: quantification of density, decision weighting according to significance, and a fusion rule that expresses the agreement among individual sensors.

## 3. Application to Experimental Data

To demonstrate the fusion technique’s performance under realistic conditions, a test specimen containing 15 surface flaws was inspected using three different NDT methods. In this section, we describe the specimen, the individual data collection and processing as well as the application of our fusion algorithm. Finally, we quantitatively evaluate the effect of various conditions on the fusion result, and compare fused detection against single-sensor detection. To further corroborate the effectiveness of our fusion approach, experiments are replicated using a second specimen. Results for this test piece are provided in the Supplementary Material to this article.

### 3.1. Specimen

**Figure 4:**
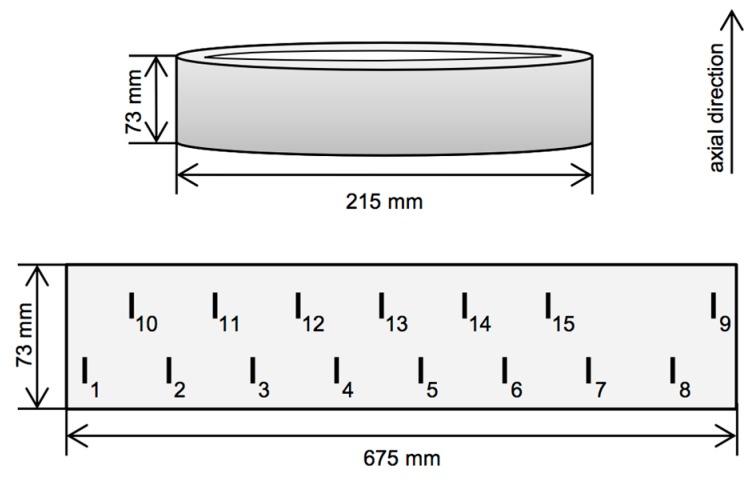
Schematic view of the ring specimen, not to scale. (**Top**) outer proportions of the ring; (**Bottom**) unrolled outer surface. Short vertical lines indicate the positions of the 15 grooves.

The ring-shaped test specimen is a bearing shell ([16], pp. 173–175) made of surface-hardened steel. As illustrated in Figure 4, it has an outer diameter of 215 mm and is 73 mm long in its axial direction. To study micro-sized elongated faults similarly to cracks, 15 grooves were inserted into the specimen by electrical discharge machining. The reason for choosing grooves over real cracks is that their dimensions can be experimentally controlled. The flaws are regularly spaced across the surface of the specimen and vary in depth from 11 to 354 µm, as detailed in Table 1. See also Table S1 in the Supplementary Materials for our second test specimen. Grooves have constant lengths of 1 mm and their openings vary between 25 µm and 51 µm. The specimen’s surface is uncoated and its roughness is very low, thus enabling high-quality near-contact measurement.

**Table 1:** Groove depths. Labels correspond to those shown in Figure 4.

Groove	1	2	3	4	5	6	7	8	9	10	11	12	13	14	15
Depth/µm	354	224	170	105	82	61	57	53	43	40	39	27	29	20	11

### 3.2. Individual Measurements and Processing

For crack detection, three different inspection techniques were used that are well-suited for the automated nondestructive evaluation of cracks in ferromagnetic metals. The first method is called *eddy current testing* (ET). An excitation coil is run over the specimen surface. Using this coil, an alternating current induces circular eddy currents in the specimen’s near-surface region. These currents are blocked by the presence of defects, thus affecting the impedance of the probe coil, which is the measured signal. The second method employed here is the laser-induced *active thermography testing* (TT). A high-power laser is run across the specimen to locally heat up the surface. In defect-free regions, the heat flow is able to dissipate, whereas defects cause localized heat accumulation. An infrared camera monitors the heat flow decay and generates a digital image sequence for processing. The third method is *magnetic flux leakage testing* (MFL). The specimen is exposed locally to a static magnetic field, which spreads inside the ferromagnetic material. At near-surface defects, the field is forced to “leak” out of the specimen into the air, although air has lower magnetic permeability than the specimen. This leakage can be detected by magnetic field sensors, such as *giant magneto resistance* (GMR) sensors. The following three inspections were performed sequentially during the course of about one year.

ET was carried out at an excitation frequency of 500 kHz, which is well-suited to inspect surface defects due to the skin effect [17]. An automated scanning device rotates the specimen under the fixed probe. Signal processing is based only on the imaginary part of the measured impedance. The obtained one-dimensional signals are preprocessed by high-pass filtering for trend correction, and by low-pass filtering to improve SNR. An image is formed by stacking the line scans in axial direction of the ring.

The MFL data were collected using the same scanner as for ET, and a GMR sensor array developed at BAM [18]. Using these gradiometers, the normal component of the magnetic stray field was measured while the specimen was locally magnetized. Preprocessing comprised trend correction by high-pass filtering per line scan, and an adaptive 2D wiener filter (We used MATLAB’s function *wiener2.* See [19]) for noise suppression. The image was then Sobel-filtered to highlight the steep flanks that are generated by the gradiometers over the grooves.

Thermography testing was performed by rotating the specimen under a 40 W powered laser while recording with an infrared camera. The movie frames were then composed to form an image of the specimen surface. This image is processed by 2D background subtraction using median filtering, and noise was suppressed by an adaptive 2D wiener filter.

We note that the presented signal acquisition is tailored to the known groove orientation. In a realistic setting, a second scan should be performed for ET and MFL testing to detect any circumferentially oriented defects as well. We further emphasize that for our specific ring specimen, the GMR sensors yield far superior results compared to ET and TT, and would suffice by themselves for surface crack detection. Specifically, the MFL data facilitate zero false alarm rate even for the second shallowest of only 20 µm depth. However, such performance is not guaranteed for other materials or in case of suboptimal surface conditions, so that a multi-method approach is still in demand. To take advantage of as many independent sources of information as possible during fusion, we intentionally lowered the quality of the MFL image before preprocessing and detection. This was done by separating the true defect indications from the background signal variations using the shift-invariant wavelet transform [20], and by reconstructing the signal with a factor of 0.02 for the noise-free component. Although this does not simulate a lower-quality MFL measurement in a physically realistic way, the approach allows making use of the acquired GMR signals as a third source of information. We justify this alteration in favor of demonstrating the capabilities of our fusion technique in other settings where individual inspection is in fact not reliable enough. Therefore, for the rest of the article, only this modified version of the MFL data is considered.

**Figure 5:**
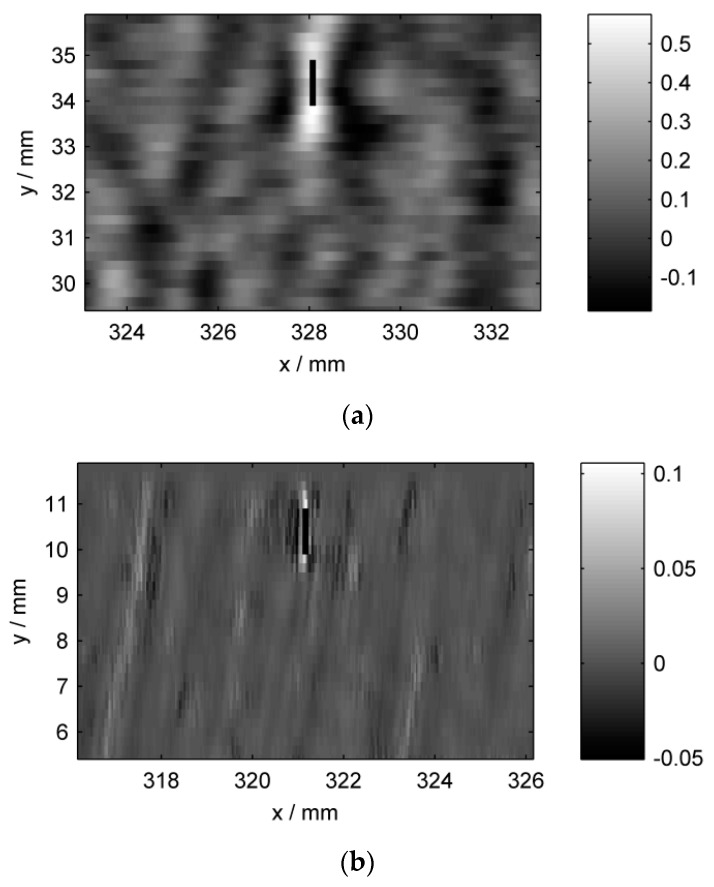

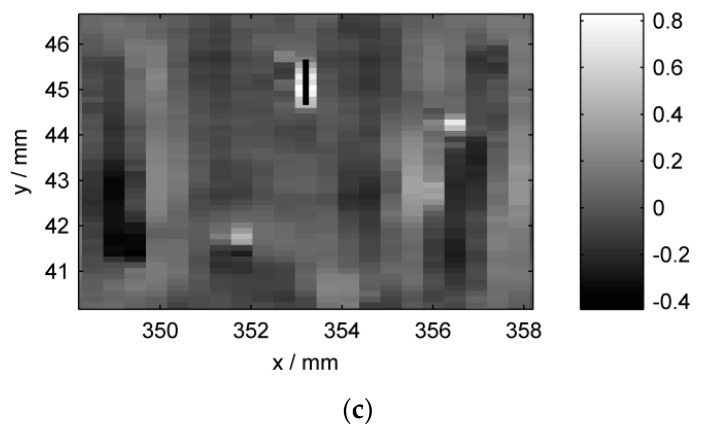
Preprocessed sensor intensity images, zoomed to a region around the groove nr. 13. Higher intensities correspond to indications. (**a**) ET; (**b**) MFL; (**c**) TT. The vertical black line marks the location of the groove. Each image is shown in the respective sensor’s coordinate system, thus explaining the different spatial axis labels.

**Figure 6:**
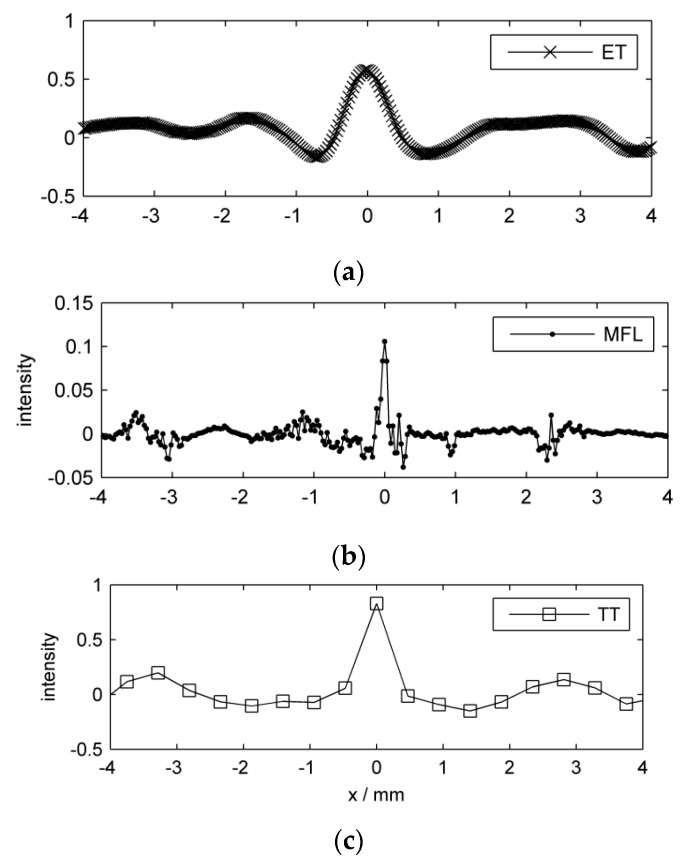
Preprocessed line scan per inspection method around groove nr. 13. (**a**) ET; (**b**) MFL; (**c**) TT. The signals are shifted so that each peak value is located at x=0. Note the different intensity scales.

To convey an impression of the signals, we show an exemplary portion of each preprocessed inspection image in Figure 5. The displayed part of the specimen surface is a 10 mm by 6.5 mm region around groove nr. 13 which is quite shallow, and thus generates relatively weak indications. The figure demonstrates the different signal patterns among sensors, concerning both the groove and the background variations. Also, the different pixel sizes are evident. A related plot is shown in Figure 6, where one-dimensional line scans crossing the groove reveal more clearly the individual sensor responses. The different spatial sampling positions are demonstrated by the line markers. Table 2 (respectively, Table S2) offers a quantitative comparison of the individual data sets.

**Table 2:** Quantitative properties of the individual data sets.

	ET	MFL	TT
$\Delta_{x}$ in mm	0.029	0.029	0.469
$\Delta_{y}$ in mm	0.200	0.200	0.126
Width of a typical indication, in mm	2 ≈ 69$\Delta_{x}$	0.6 ≈ 20$\Delta_{x}$	0.5 ≈ $\Delta_{x}$
Avg. nr. of hits per pixel	0.0023	0.0031	0.0068

After individual preprocessing, the same detection routine was used for all three images to extract hit locations and confidences, which will later be fused at the decision level. To this end, we convert the signal intensities to confidence values and apply a threshold to extract only significant indications, as follows. Confidence values are computed by estimating the distribution function of background signal intensities from a defect-free area. This estimate serves as the null distribution $p_{null}\left(x\right)$ in the significance test. For each image pixel’s intensity y, the probability $p_{null}\left(y\geq x\right)$ is computed as the confidence. Pixels are considered significant here, if their confidence exceeds 99%. Additionally, only those hits that are local maxima with regard to their neighboring pixels along the horizontal axis (thus crossing the grooves) are retained. This constraint further filters many false alarms while making the detection results invariant to different peak widths. After detection, each hit is associated with its local signal to noise ratio, which will be used as the weight $w_{d}$ during density estimation according to Equation (2). To this end, we set $w_{d}=\left(y-E\left(X\right)\right)/Std\left(X\right)$. That is, the image intensities y are standardized with regard to the null distribution of background signal intensities X for each sensor.

**Figure 7:**
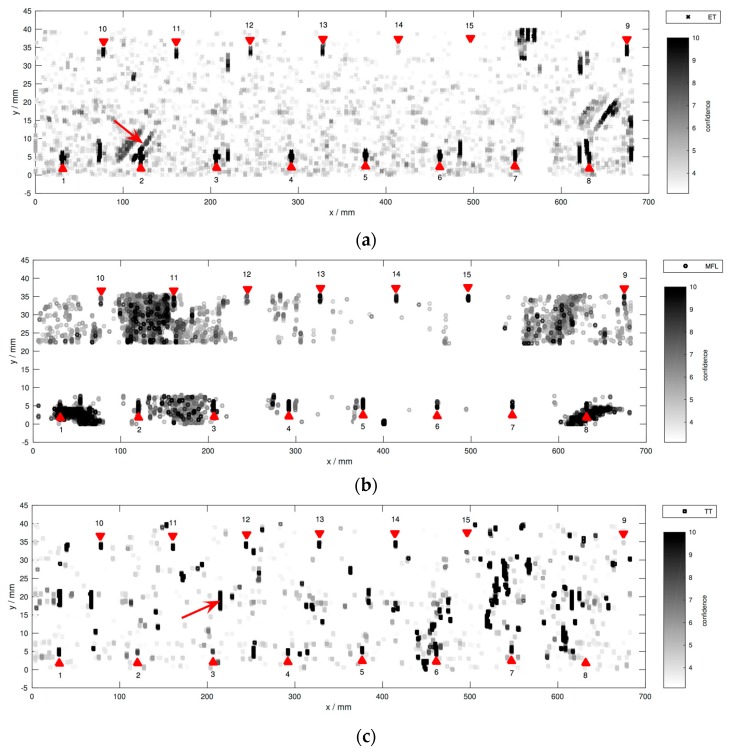
Hit locations per sensor in a common coordinate system similar to that in Figure 4. (**a**) ET; (**b**) MFL; (**c**) TT. Darker colors correspond to higher SNR. Note that the color scale is clipped to a maximum of 10 to prevent non-groove hits from dominating. Axes *x* and *y* are not to scale. The tips of the triangular markers indicate the groove positions. The two arrows point to prominent crack-like indications (false positives) in the ET and TT images.

After registration to a common coordinate system based on manually defined location correspondences in the data, the final set of hits from all sensors is plotted in Figure 7. Obviously, the false alarms considerably outnumber the actual groove hits. This is due to our sensitive detection rules, intending that no actual defect is missed during individual processing. 

Of course, in a single-sensor inspection task, a much more stringent detection criterion is appropriate to limit the number of false alarms. However, this possibly leads to worse sensitivity to small flaws. In contrast, our data fusion approach is supposed to discard most false hits while maintaining high sensitivity to small defects.

We will now briefly compare the individual sensor results. In contrast to ET and TT, the MFL hits cluster spatially. This is because the background variations in this data set are not homogeneous, possibly due to inhomogeneities in the internal magnetic field. MFL data are missing in the strip between the two groove rows. The shallowest groove nr. 15 features low SNR in the ET and TT data due to its shallowness. MFL in contrast is more sensitive. Moreover, grooves nr. 8 and 9 stand out in the TT data, because their confidences are even weaker than the shallower grooves nr. 10–14. Interestingly, in Figure 7, spatial defect-like patterns are formed, although the specimen is not expected to contain any flaws other than the known grooves. For example, see the vertical lines from TT, or the diagonally oriented lines from ET, as highlighted by the arrows. As previously discussed, using individual inspection, it is not easy to classify these obvious indications as structural indications or flaws. In spite of their regular structure, we label these patterns as non-defect indications during the following evaluation, if our multi-sensor data set is not able to give a reliable confirmation. On the other hand, there are a few off-groove locations where different sensors behave consistently. These regions could in fact represent unknown but real defects, and will therefore be excluded from the following evaluation. Note that hits within disregarded areas are not shown in this figure. Moreover, the confidence associated with each hit is not shown, because all hits exceed the chosen threshold of 99% as explained before.

### 3.3. Fusion and Final Detection

**Figure 8:**
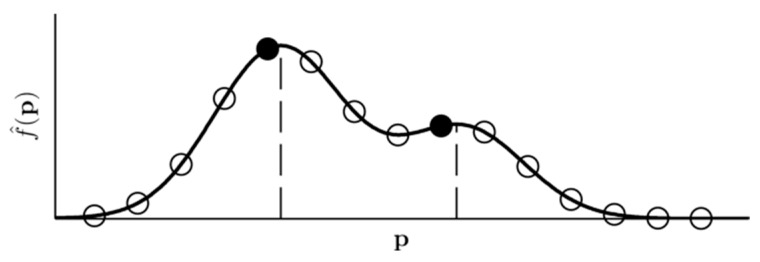
Final detection by evaluating the fused continuous density function at gridded points (circles) and finding local maxima (filled circles) among them. These hits approximate the true density modes (dashed lines).

To compute the kernel density per sensor, we used Alexander Ihler’s KDE Toolbox for MATLAB [21]. The fused density, according to Equation (3), is a continuous function that must be evaluated at discrete locations. In fact, to circumvent the discrete sampling, a multivariate mode-seeking algorithm could be used for detection. However, for simplicity, we set up a discrete evaluation grid that is designed fine enough to not miss any mode of the density. Modes are then traced similarly to per-sensor detection by finding local density maxima along parallel lines on the specimen surface. Figure 8 displays the principle of the detection. Finding local maxima along one-dimensional lines is straightforward due to the density’s smoothness, and also makes the detection results more stable across different kernel sizes.

**Figure 9:**
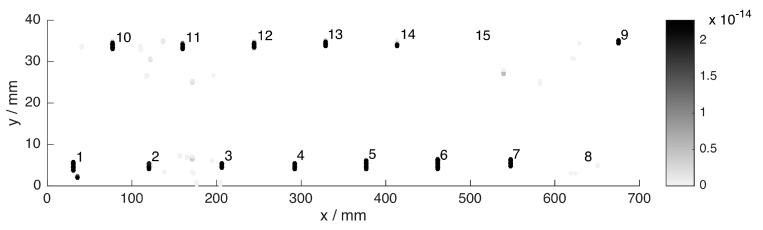
Result of decision level fusion. Darker markers correspond to increased detection confidence. The colors are scaled so that white represents zero fused intensity, and black corresponds to intensities at least as large as at the shallow defect nr. 14. Axes *x* and *y* are not to scale.

The final hits after fusion are presented in Figure 9, where fusion is performed according to Equation (3) and the *product* fusion rule. Most of the single-sensor false alarms from Figure 7 were discarded by our fusion method by recognizing the sensor conflicts. Yet, there are a considerable number of remaining false hits. These spurious hits originate from single-sensor hits that overlap purely by chance. Nevertheless, all grooves but the shallowest, nr. 15, clearly stand out against the false alarms considering the fused density measure, which is represented by the marker colors in Figure 9. Because higher values of the fused measure correspond to increased defect likelihood, a threshold can be applied to produce a binary decision. In the following, the detection performance will be quantitatively assessed under various conditions.

### 3.4. Evaluation

In the following sub-sections, our fusion method is quantitatively evaluated with regard to the presented specimen. This evaluation focuses on detectability, meaning the ability to distinguish between grooves and background in the fusion result. Consequently, the ability to accurately localize a defect after fusion is not a part of this evaluation. For each detection result in the next sections, indications are assigned fuzzy membership values to the two sets “defect” and “non-defect”, based on their distances to the known groove locations. Using this ground truth information, evaluation is carried out automatically by means of precision-recall-curves. Similarly to conventional Receiver Operating Characteristic (ROC) analysis [22], which is based on recall = true positive rate = *#true hits*/*#max possible true hits* and false alarm rate = *#false hits*/*#max possible false hits* for each possible detection threshold, we replace false alarm rate with precision = *#true hits*/*#all hits*. This choice is necessitated by the scattered nature of the hits, which allow an infinite number of possible false alarms, that is off-groove locations. Precision circumvents this restriction by relating hits to hits, rather than hits to non-hits.

The two evaluation measures precision and recall are fuzzyfied in our evaluation to include the fuzzy membership per hit in the analysis ([23], p. 46). That is, each hit is allowed to be counted partially as a true positive and as a false alarm: Indications near known groove locations are evaluated nearly 100% as true positives, whereas hits that lie further away have an increasing share as a false alarm. The correspondence between distance to the nearest groove location and fuzzy membership is realized by a Gaussian membership function, whose spread parameter σ=0.2 mm is set equal to the estimated mean registration error for our data set to account for the localization uncertainty.

Once an evaluation curve in fuzzy ROC space per detection method and per groove is established, the area under each precision-recall-curve quantifies detection performance over the full range of detection thresholds. However, we do not compute the area under the whole curve, but only for the curve region where recall >0.5. We denote this measure by AUC-PR-0.5. This focuses our evaluation on thresholds that are low enough to ensure that at least half of a groove is detected. Furthermore, a single false alarm hit with higher intensity than the groove suffices to force the curve down to zero precision for small true positive rates, *i.e.*, high thresholds, and therefore dominates the whole AUC measure. This is another reason for ignoring the lower half of the diagram in the computation of AUC-PR-0.5.

Several regions on the specimen surface are marked to be excluded from the evaluation. These are areas near the border of the specimen, indications that result from experimental modification of the specimen surface and off-groove areas where real unplanned defects exist (which would otherwise be counted as false alarms). Not only are all of these disregarded regions removed from evaluation after fusion, but already the hits in these regions are excluded from the density estimation, so that they don’t affect the density in the surrounding regions. Furthermore, to evaluate detection performance per flaw depth, after fusion each groove is assessed individually while ignoring all others.

All fusion results are evaluated at the same locations on the specimen surface defined by a dense grid with sampling distances $\Delta_{x}=0.0289\;\text{mm},\Delta_{y}=0.1258\;\text{mm}$. This choice of grid resolution is given by the finest spatial sampling among all individual sensors in each spatial dimension. Indications are found by local maximum detection as described in Section 3.3.

If not stated otherwise, fusion is carried out with a fixed kernel size per sensor according to Equation (4), using $\hat{u}=0.2\;\text{mm}$. For example, the ET sensor is assigned a kernel size of $\left(h_{x},\;h_{y}\right)=\left(0.2\;\mathrm{mm},\;1.3793\;\mathrm{mm}\right)$. While this automatic formula ensures that the kernel size exceeds the localization uncertainty in both spatial dimensions and retains the ratio of sampling distances, it might lead to situations in which the kernel is extremely large in the coarsely sampled direction, as is seen here: The kernel size in the y direction is even larger than the 1 mm long grooves themselves, which results from the disproportionate sampling distances in our measurements (see Table 2). To avoid introducing unrealistically large kernels, we restrict the kernel size ratio to at most 3. Consequently, for ET and MFL data, the kernel sizes $\left(h_{x},\;h_{y}\right)=\left(\hat{u},\;3\hat{u}\right)=\left(0.2\;\mathrm{mm},\;0.6\;\mathrm{mm}\right)$ are applied. Using this evaluation framework, we investigate the performance of our fusion approach in the following.

#### 3.4.1. Evaluation of Fusion Rules

As described in Section 2.2.3, the three normalized per-sensor densities are fused at each location of interest on the specimen surface using some fusion rule. In this study, we compare the following eight functions: *minimum*, *geometric mean*, *harmonic mean*, *product*, *median*, *sum*, *sumIgnoreMax* according to Equation (7), and the *maximum*. These are contrasted with single-sensor performance, both before and after individual kernel density estimation. All single-sensor hits are assessed here, in contrast to the fusion methods where hits below the per-sensor thresholds were discarded. For each groove, a separate ROC analysis was carried out to assess the influence of defect size.

At this point, we emphasize that the results presented in this article are not representative for the general performance of each individual inspection method. It is possible that better individual results than shown here may be obtained by optimizing e.g., the specimen preparation, the sensors or the processing routines. This is especially true for the MFL data, which were artificially degraded as described in Section 3.2. Rather, in the following our focus is to demonstrate that our technique is able to perform well in the face of imperfect sources of information.

**Figure 10:**
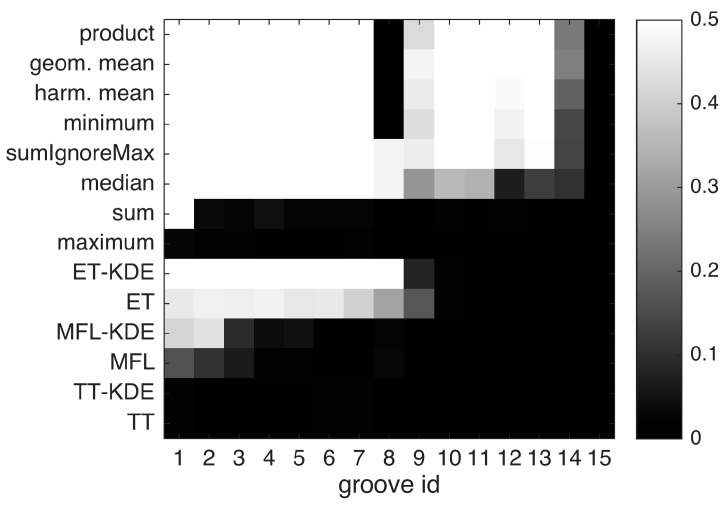
Evaluation of different fusion functions F according to Equation (2), and of single-sensor detection. For each groove and detection method, the AUC-PR-0.5 is shown in shades of gray. Optimal performance is 0.5. Groove numbers correspond to Table 1, that is groove nr. 1 is the deepest and nr. 15 is the shallowest.

The results are presented in Figure 10. In agreement with the visual impression from Figure 7, single-sensor performance (ET, MFL and TT) is unsatisfactory. Although the individual KDEs better pronounce the grooves against more randomly scattered background hits, false alarms still prevent reliable detection even for deep grooves (see TT-KDE and MFL-KDE). After purposely degrading the MFL data (see Section 3.2), the eddy current technique provides the best single-sensor detection results by reliably indicating groove depths no less than 55 µm (groove nr. 8). In contrast, through multi-sensor fusion, most of the defects can be detected reliably. Two exceptions are the *sum* and the *maximum* rule, which perform poorly over most if not all grooves. These results are explained by the fact that *sum* and *max* do not quantify agreement among sensors, but instead retain all indications from any individual sensor in the fusion result. This is prone to false alarms, which is reflected by low evaluation scores. In contrast, the *minimum*, *geometric* and *harmonic mean* and the *product* rule yield high scores for most defects. Apparently, grooves nr. 8 and 9 are hard to identify across many fusion methods despite the grooves’ midsize depths. This suggests poor single-sensor SNR at these locations, thus leading to inter-sensor conflict, so that this groove is wrongly classified as a false alarm by the strict fusion methods *product*, *geometric* and *harmonic mean* and *minimum*. On the other hand, the milder fusion rules *median* and *sumIgnoreMax* tolerate unknown single-sensor dropout at the expense of comparably poor detection performance at the shallowest grooves. Specifically, whereas the *median* rule might deteriorate in the face of overall low SNR by permitting too many false alarms, *sumIgnoreMax* offers a good compromise between strictness and tolerance in our evaluation. However, for the detection of very shallow defects like groove nr. 14 in our specimen, stricter rules appear to offer better performance. The best fusion rule in this evaluation is the *geometric mean*, closely followed by the *harmonic mean* and the *product*. As *product* is conceptually extremely simple yet effective, it is considered the winner. Overall, the shallowest detectable groove depth in our study is given by 29 µm at groove nr. 13. The 20 µm groove nr. 14 could not be found reliably, although fusion offers improved detectability compared to single-sensor detection. The shallowest groove nr. 15 (11 µm) is not distinguishable from background noise due to lack of single-sensor sensitivity.

**Figure 11:**
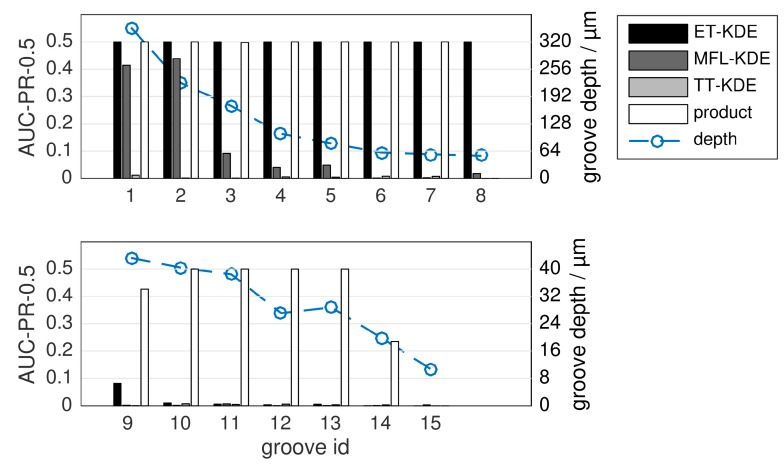
Evaluation of single-sensor detection (ET-KDE, MFL-KDE, TT-KDE) *versus* fusion, for the *product* fusion rule and a fixed kernel size. The maximum possible score is 0.5 (left vertical axis). The set of grooves is divided into two sub-figures for clarity. Groove depth is indicated by the blue line corresponding to the right vertical axis. Note the different axis scales for groove depth in the two subplots.

Additionally, the results are more clearly presented in Figure 11, where only the *product* fusion is compared against the single-sensor KDEs. This experiment was also re-produced using a second test specimen, which we report in Figure S1 in the Supplementary Material to this article.

#### 3.4.2. Influence of Kernel Size

**Figure 12:**
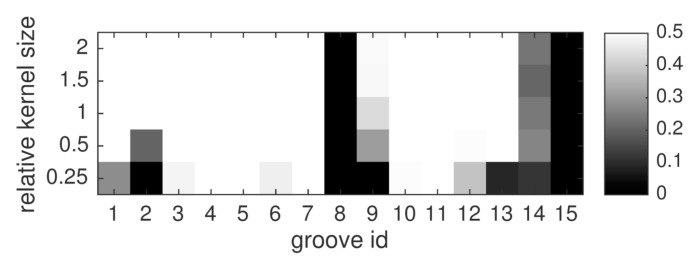
Evaluation of different kernel sizes, for the *product* fusion rule. For each groove and fusion method, the AUC-PR-0.5 is shown in shades of gray.

Just like in conventional kernel density estimation, the kernel size is an important factor regarding the detection performance. The sizes assessed in this study are arranged in Table 3. The *product* fusion rule is selected here due to its strong performance in the previous experiment. Evaluation results are shown in Figure 12. The results suggest that given a well-performing fusion method and an accurate estimation of the localization uncertainty $\hat{u}$, a range of kernel sizes around the proposed default setting in Equation (4) is adequate. Performance only deteriorates for very small kernel sizes like 25%–50% of the proposed size. The product rule shows no obvious dependence between kernel size and performance at the shallowest two grooves. However, groove nr. 9 is better identified when larger kernels are used. This could be explained by unusually large registration error at this region, but in this case the reason is that thermography only indicates the top part of groove nr. 9 with large enough SNR to pass the individual detection stage. The results presented here might tempt to favor large kernels. However, large kernels increase the chance of falsely associating spatially nearby false alarms, and thus quantify sensor agreement where there is actually conflict. Therefore, the kernel size proposed in Equation (4) was found to be effective in this experiment.

**Table 3:** Kernel sizes used in the experiment. All sizes are in mm. *Relative kernel size* denotes the fraction of $\hat{u}=0.2\;\text{mm}$ that was used to compute the kernel sizes according to Equation (4). That is, a range of smaller and larger kernels compared to the default size (relative kernel size = 1, bold faced column) were assessed. To prevent unrealistically large kernels due to the disproportionate spatial sampling distances in our data, kernel sizes were limited to 0.6 mm for ET and MFL, and to 0.75 mm for TT (gray shaded table cells).

		Relative Kernel Size				
		0.25	0.5	1.0	1.5	2
ET	$h_{x}$	0.05	0.1	**0.2**	0.3	0.4
	$h_{y}$	0.346	0.6	**0.6**	0.6	0.6
MFL	$h_{x}$	0.05	0.1	**0.2**	0.3	0.4
	$h_{y}$	0.345	0.6	**0.6**	0.6	0.6
TT	$h_{x}$	0.186	0.373	**0.746**	0.746	0.746
	$h_{y}$	0.05	0.1	**0.2**	0.3	0.4

#### 3.4.3. Influence of Weights

**Figure 13:**
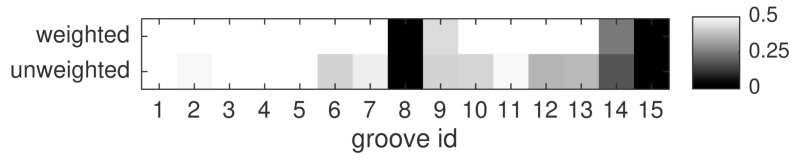
Comparison between the weighted approach (as proposed; top row) and the unweighted variant, for the *product* fusion rule.

In the previous experiments, the individual sensors’ hits were weighted by factors $w_{d}$ in Equation (2) to take into account the local SNR. This experiment assesses the benefit of these weights over an unweighted approach ($w_{d}=1$) in which the densities ${\hat{f}}_{S}\left(p\right)$ are only influenced by the spatial proximity of neighboring hits. This setting represents inspection results for which no measure of confidence is available. In both experimental cases, the *product* fusion rule was chosen and the kernel size was set to the value suggested by Equation (4). Figure 13 illustrates the respective detection performances. According to the results, the unweighted variant never surpasses the proposed weighted density estimation at any groove. Interestingly, although the unweighted method does not take into account the local SNR and therefore is not influenced by defect depth, it is clearly observed that most of the deeper grooves (e.g., nr. 1–5) are more reliably found than the shallower grooves (e.g., nr. 9–15). This is because during the first stage of individual detection before fusion, only parts of the shallower grooves might be retained whereas deeper grooves are completely preserved. Therefore, the weighted approach should be favored over the unweighted method if possible. Otherwise, much effort should be spent on high-quality registration to make the sole feature of spatial proximity of hits across different sensors a reliable indicator of defect presence. Yet, even without weights, *product*-fusion still outperforms any individual method in our evaluation for grooves shallower than 43 µm (groove nr. 9).

#### 3.4.4. Influence of Individual Sensors

**Figure 14:**
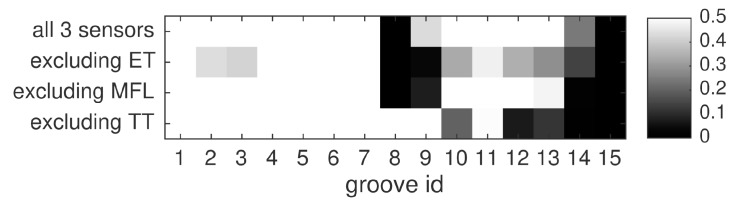
Influence of individual sensors on the fusion result, for the *median* fusion rule and a fixed kernel size.

The effect of individual sensors on the fused performance is assessed in this study. To this end, fusion is carried out three times, while leaving out the hits of one of the sensors in each run. The results are compared to fusing the full data set. Again, the *product* fusion rule is applied, and hits are weighted. The results are presented in Figure 14. Each two-sensor subset of inspection methods shows slightly different effects. Apparently, the thermographic data mainly help in detecting the shallow grooves 12–14. However, the same inspection seems to have missed the flaws 8 and 9, because the information from both MFL and ET is crucial for detection here. On the other hand, TT is required to identify most of the shallow grooves in this evaluation. The same observation holds for ET. In contrast, by purposely degrading the MFL data (see Section 3.2), we reduced the positive effect this inspection method has on multi-sensor detection. Still, although this low-quality data has a large set of false alarms, it impairs multi-sensor detection for none of the grooves. On the contrary, MFL improves the detection quality of grooves 9, 13 and 14. Among the deeper grooves, nrs. 1, 4, 5, 6 and 7 are perfectly found using any two-sensor configuration, thus indicating that they are clearly represented in all three measurements. Overall, the evaluation demonstrates that the full set of sensors is required for optimal performance with our data set. Yet, with the right choice of sensors two-source fusion already has the potential to outperform individual detection.

## 4. Discussion

Our experiments demonstrate that our density-based approach is well-suited to incorporate indications from heterogeneous sensors. The number of false alarms can be strongly reduced relative to single-sensor inspection while retaining most of the defects. Regarding our evaluation index, for 13/15 grooves, the fusion method performs as well as or better than single-sensor detection. The performance gain is most pronounced for the shallower defects, which usually generate less significant indications.

We note that the principle to quantify agreement among sensors requires that all sensors yield redundant information about the object of interest, e.g., near-surface cracks in our case. That is, in NDT, all sensors must respond to the same flaw type in the same size range. If, in contrast, one of the sensors reports a defect that is not detectable by the other methods, it will be discarded as a “false alarm” by our technique, since it is not designed to fuse complementary information. This is the case for the shallowest investigated defect in the study. Similarly, groove nr. 8 is only found by single-sensor inspection but not by fusion, because TT lacks of a significant indication in that area. To apply a multi-sensor system despite such unexpected effects, mild fusion rules, such as *median* or *sumIgnoreMax*, trade strong reduction of false alarms for the ability to compensate unknown sensor dropout.

Another point concerns the relationship between fusion performance and spatial uncertainty. Specifically, the fusion performance is expected to improve with registration accuracy. This is because kernels can be made narrower for smaller registration errors, and therefore the likelihood of a non-defect-related indication due to spurious multi-sensor agreement is reduced. In any case, the actual registration error must be quantified to set the kernel size accordingly. Moreover, the fusion technique strongly benefits from realistic estimates of the local signal to noise ratios, which enter the fused density through weights. For the final detection after fusion, a threshold could be chosen to retain only the significant density peaks. Thus, only few parameters (localization uncertainty, fusion rule, density threshold) fully describe the methodology and are usually readily determined. Furthermore, if one is unsure about a fused indication, the original individual hits can always be reconsidered to collect additional evidence for or against the presence of a defect. After all, the density-based approach spatially associates neighboring hits and thus may serve as the basis for multi-sensor detection after feature extraction. For example, our density measure identifies narrow regions of increased defect likelihood. These regions can further be assessed by extracting features from each individual sensor in this region, which could be combined by some classification algorithm to reach a final conclusion.

Concerning the number of sensors, we suggest using at least three different sources of information, as presented here. However, our experiments show that improved performance over single-sensor inspection is possible already for two sensors. Note that the more fusion inputs are provided, the higher the likelihood of the purely coincidental agreement between at least two sensors. Therefore, the rule *sumIgnoreMax* (Equation (7)) will have to be extended if even more sensors are included, whereas the *median* rule and *product* rule is expected to perform well regardless of the number of sensors.

We would also like to point out the limitations of the current study. Whereas the eroded grooves facilitate detection assessment for well-defined defect depths, their linear shapes do not resemble natural defects. Also, whereas the orientation of our flaws is well-defined, natural defects often vary in orientation. Therefore, directionally sensitive measurements, such as ET using a differential probe and MFL using gradiometers, must be carried out multiple times in different directions. However, because this issue is only relevant for per-sensor detection prior to fusion, it is not further elaborated here.

## 5. Conclusions/Outlook

We developed a density-based method for the fusion of spatially scattered data and applied it to sensor signals from the nondestructive testing of a bearing shell. This high-level fusion approach has the advantage of being independent from the processes that generate the scattered points, and of directly accounting for registration errors. Three different mechanisms are implemented to ensure robustness against false alarms. Practical suggestions on how to determine the kernel size are given. We quantitatively evaluated our technique using a defect detection experiment. The results demonstrate that single-sensor inspections of the specimen are outperformed by the proposed technique, especially for defects that are too shallow to be reliably indicated otherwise. Moreover, the proposed method is quite generic, as it receives spatial locations from single-source detection routines and returns areas of multi-sensor agreement. Therefore, it may be applied for detection tasks in other domains, such as multi-modal medical image fusion.

## Supplementary Materials

The following are available online at www.mdpi.com/1424-8220/16/1/105/s1.
