# Supplementary material for: Decision-Level Fusion of Spatially Scattered Multi-Modal Data for Nondestructive Inspection of Surface Defects
Source: Sensors (Basel). 2016 Jan 15;16(1):105. doi: 10.3390/s16010105 (PMC4732138; doi:10.3390/s16010105)
Supplement: Supplementary file 1 [file sensors-16-00105-s001.pdf]

Supplementary Materials: Decision-Level Fusion of Spatially Scattered Multi-Modal Data for Nondestructive Inspection of Surface Defects

René Heideklang and Parisa Shokouhi

Results Obtained for a Second Test Specimen

In addition to the specimen discussed in the main article, denoted by SA, the presented fusion approach is applied to a second specimen (SB) to demonstrate the transferability of our results to other samples.

The second investigated test specimen is identical to the first bearing shell, thus having the same physical and geometrical properties such as constituent material, shape, size and surface condition. SB also contains regularly spaced machined grooves simulating surface cracks. But whereas SA contains 15 grooves ranging in depth from 10 to 385 µm, SB has 16 grooves in a narrower range of between 10 and 50 µm. The detailed specifications of SB grooves are given in Table S1.

Table S1. The depths of grooves in specimen *SB*. For reference, grooves of comparable depth in *SA* are listed in the last row.

**Groove nr.**	**1**	**2**	**3**	**4**	**5**	**6**	**7**	**8**	**9**	**10**	**11**	**12**	**13**	**14**	**15**	**16**
Depth/µm	54	54	35	33	31	31	28	28	26	24	22	20	19	15	14	12
Comparable groove nr. in *SA*	8		11				13			14					15	

Data collection was carried out as described in the main article. However, the analysis differs from that of specimen SA in the following aspects:

- The spatial sampling distances differ, as detailed in Table S2. Specifically, sampling is finer during the inspection of SB by both eddy current and thermal testing. Consequently, the grid where the fused densities are evaluated is refined to $\Delta_x = 0.0288\text{ mm}, \Delta_y = 0.100\text{ mm}$, which equals the grid resolution of ET.
- Several regions on the surface of SA were identified that had to be excluded from evaluation for reasons given in the main article. In contrast, no region was excluded from the evaluation of SB.
- The mean registration error of the inspections of SB is slightly higher (around 0.25 mm) than for SA (around 0.2 mm).
- Due to the changes in localization uncertainty and in spatial sampling distances, new kernel sizes suggested by Equation (4) were applied. As for SA, kernel bandwidth parameters (h_x, h_y) were restricted to have a ratio of at most 3. For ROC evaluation, the fuzzy membership parameter was set to $\sigma = 0.2\text{ mm}$ as for SA.
- To degrade the quality of the MFL data set for a meaningful assessment of fusion performance, the sensor indications were reduced to 20% of their original intensities, in contrast to 0.2%. This setting produces roughly comparable signal to noise ratios in SA and SB at shallow grooves.

Table S2. A comparison of spatial sampling distances during the inspection of specimens *SA* and *SB*. Dissimilar distances for *SB* are in boldface. All measures are in μm.

	SA	*SB*
ET	28.9 × 200	28.8 × **100**
MFL	29 × 200	28.9 × 200
TT	469.1 × 125.8	**125.6** × 125.6

Evaluation of Fusion Rules

The same eight fusion rules applied to the measurements on *SA* are quantitatively compared against single-sensor detections for *SB*. Figure S1 presents the results. The results are consistent with those obtained from the first specimen *SA*. Fusion outperforms single-sensor detection in all cases, except for MFL at groove nr. 9. Defect nr. 14 demonstrates the advantage of strict rules (e.g., product) over less strict rules (e.g., median) to reliably identify shallow defects. Grooves 8 and 9 are hard to find across many detection methods due to poor single-sensor SNR and, in the case of groove nr. 9, due to an unusually large local registration error of 0.75 mm. Note that the mean registration error is about 0.25 mm. The shallowness of grooves nr. 13 and above (shallower than 20 μm) results in an insufficient single-sensor SNR. Yet, groove nr. 14 appears to yield relatively strong indications in the data, which is additionally aided by low local registration error (about 0.2 mm). Whereas in *SA*, the geometric mean is slightly ahead of harmonic mean and product, in *SB* the harmonic mean takes the first place, followed by geometric mean and product. Again, the product rule can be considered the most basic method that performs best.

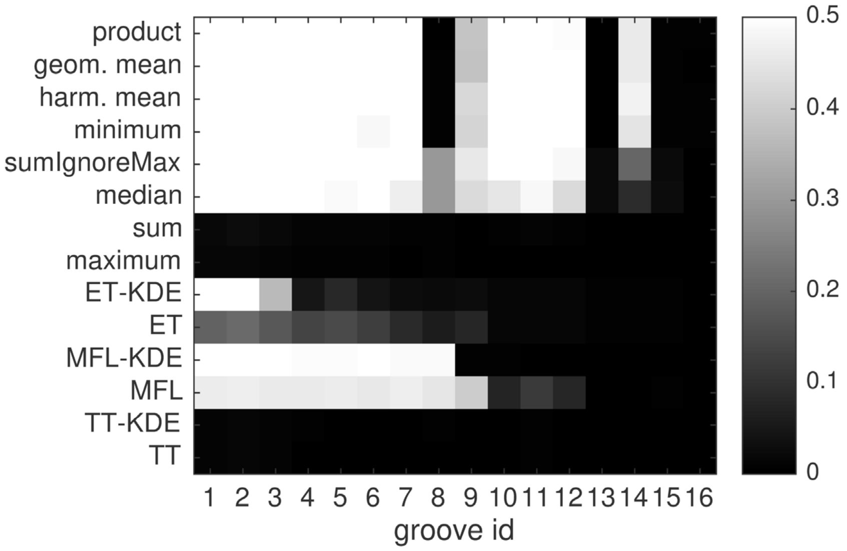

Figure S1. Evaluation of different fusion functions F according to Equation (2), and of single-sensor detections. For each groove and detection method, the AUC-PR-0.5 is shown in shades of gray. Optimal performance is 0.5. Groove numbers correspond to those given in Table 4, that is groove nr. 1 is the deepest and nr. 16 is the shallowest.
